# Bronchus sign on thin-section computed tomography is a powerful predictive factor for successful transbronchial biopsy using endobronchial ultrasound with a guide sheath for small peripheral lung lesions: a retrospective observational study

**DOI:** 10.1186/s12880-015-0060-5

**Published:** 2015-06-21

**Authors:** Tomoyuki Minezawa, Takuya Okamura, Hiroshi Yatsuya, Naoki Yamamoto, Sayako Morikawa, Teppei Yamaguchi, Mariko Morishita, Yoshikazu Niwa, Tomoko Takeyama, Yuki Mieno, Tami Hoshino, Sakurako Uozu, Yasuhiro Goto, Masamichi Hayashi, Sumito Isogai, Masaki Matsuo, Toru Nakanishi, Naozumi Hashimoto, Mitsushi Okazawa, Kazuyoshi Imaizumi

**Affiliations:** Division of Respiratory Medicine and Clinical Allergy, Department of Internal Medicine, Fujita Health University, 1-98 Dengakugakubo, Kutsukake-cho, Toyoake, Aichi 470-1192 Japan; Department of Public Health, Fujita Health University, Toyoake, Aichi Japan; Laboratory of Molecular Biology & Histochemistry, Fujita Health University, Toyoake, Aichi Japan; Department of Respiratory Medicine, Nagoya University Graduate School of Medicine, Nagoya, Japan; Department of Respiratory Medicine, Chubu Rosai Hospital, Nagoya, Japan; Department of Respiratory Medicine, Daiyu-kai Hospital, Ichinomiya, Aichi Japan

**Keywords:** EBUS-GS, CT bronchus sign, Diagnostic yield, Thin-section CT, Peripheral lung lesion

## Abstract

**Background:**

Recent advances in bronchoscopy, such as transbronchial biopsy (TBB) using endobronchial ultrasonography with a guide sheath (EBUS-GS), have improved the diagnostic yield of small-sized peripheral lung lesions. In some cases, however, it is difficult to obtain adequate biopsy samples for pathological diagnosis. Adequate prediction of the diagnostic accuracy of TBB with EBUS-GS is important before deciding whether bronchoscopy should be performed.

**Methods:**

We retrospectively reviewed 149 consecutive patients who underwent TBB with EBUS-GS for small-sized peripheral lung lesions (≤30 mm in diameter) from April 2012 to March 2013. We conducted an exploratory analysis to identify clinical factors that can predict an accurate diagnosis by TBB with EBUS-GS. All patients underwent thin-section chest computed tomography (CT) scans (0.5-mm slices), and the CT bronchus sign was evaluated before bronchoscopy in a group discussion. The final diagnoses were pathologically or clinically confirmed in all studied patients (malignant lesions, 110 patients; benign lesions, 39 patients).

**Results:**

The total diagnostic yield in this study was 72.5 % (95 % confidence interval: 64.8–79.0 %). Lesion size, lesion visibility on chest X-ray, and classification of the CT bronchus sign were factors significantly associated with the definitive biopsy result in the univariate analysis. In the multivariate analysis, only the CT bronchus sign remained as a significant predictive factor for successful bronchoscopic diagnosis. The CT bronchus sign was also significantly associated with the EBUS findings of the lesions.

**Conclusion:**

Our results suggest that the CT bronchus sign is a powerful predictive factor for successful TBB with EBUS-GS.

## Background

Recent studies of lung cancer screening by low-dose computed tomography (CT) have demonstrated a definite advantage for detecting early-stage lung cancer [1–3]. Both chronic obstructive pulmonary disease and pulmonary fibrosis, rapidly growing health problems especially in the older population, are frequently associated with lung cancer [4, 5]. In patients with such diseases, a chest CT scan is essential for disease evaluation or monitoring. An increasing number of small-sized peripheral lung lesions are being found incidentally during the follow-up of these diseases [6]. Accordingly, there is an urgent need for the development of strategies to confirm the pathological diagnosis of small-sized lung lesions. Bronchoscopy has been the mainstay diagnostic method for pulmonary lesions. Recent advances in newer bronchoscopic techniques, such as endobronchial ultrasound (EBUS) or the use of virtual navigation systems, have enabled us to approach small peripheral lung lesions that are invisible on fluoroscopy [7]. However, recent studies have shown that a final diagnosis cannot be reached by bronchoscopy even with the most advanced techniques available today in a certain percentage of patients [8].

Recent developments in molecular targeted therapy for lung cancer have increased the need to obtain bronchoscopic biopsy specimens sufficient for molecular analysis [9]. Various diagnostic procedures other than bronchoscopy, such as CT-guided percutaneous needle aspiration biopsy (CTNAB) and surgical biopsy, are also available. Physicians must choose the most suitable procedure with which to establish a diagnosis in patients who present with lung lesions. It is very important to predict the possibility of obtaining an adequate bronchoscopic tissue specimen for pathological and molecular diagnosis. Based on the present analysis, CT bronchus sign on thin-section CT (TSCT) at the initial evaluation seems to be the most reliable predictive factor for successful biopsy by bronchoscopy. The results of this study provide both patients and doctors with useful information that will allow for informed consent in the selection of a diagnostic procedure.

## Methods

### Patients

The medical records of consecutive patients who underwent bronchoscopy in Fujita Health University Hospital from April 2012 to March 2013 were retrospectively reviewed. The institutional review committee (Fujita Health University Institutional Review Board) approved this study protocol, which was conducted in accordance with the tenets of the Declaration of Helsinki (approval number Fujita 14-152). All patients provided written informed consent before undergoing bronchoscopy.

### Evaluation before bronchoscopy

All patients underwent chest TSCT (0.5 mm slice) within 1 month before bronchoscopy. TSCT images were generated using a nonenhanced multidetector CT system (Aquilion One Vision Edition; Toshiba Medical Systems, Tokyo, Japan). The CT scan parameters were as follows: tube current, automatic exposure control SD7; tube voltage, 120 kV; rotation speed, 0.5 s/rot; table speed, BP 1.39; reconstruction filter, FC51 AIDR 3D WEAK; window width, 1600; and window level, −600. We used automatic exposure control, the volume helical scan mode, and multi-planar reconstruction images.

**Fig. 1:**
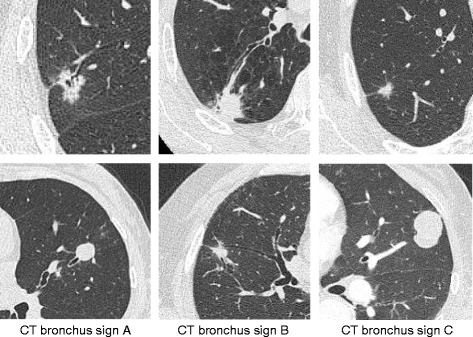
CT bronchus sign. According to TSCT (0.5-mm reconstruction), we categorized the CT bronchus sign in each case into three types **a** to **c** according to the relationship between the nearest bronchus and the target lesion. In type *A*, the responsible bronchus clearly reached the inside of the target lesion. In type *C*, no bronchus could be detected in relation to the lesion. When the CT findings could be categorized into neither type *A* nor *C*, the CT bronchus sign was categorized as type *B*

We did not use a virtual bronchoscopic navigation system. Each bronchoscopist studied the patient’s chest X-ray and TSCT images before the procedures and identified the size and location of the lesions and the respective bronchus. In every case, we confirmed these findings and identified the target bronchus to approach by group discussion. We investigated the bronchus sign on CT, which was identified as the presence of a bronchus directly leading to the target lesion (CT bronchus sign) [10]. In each case, we categorized the relationship between the target lesion and the nearest bronchus into three types of CT bronchus signs (A to C). In type A, the responsible bronchus clearly reached the inside of the target lesion. In type C, no bronchus could be detected in relation to the lesion. When the CT findings could be categorized into neither type A nor C, the CT bronchus sign was categorized as type B (Fig. 1). Two bronchoscopists (T.M. and K.I.) independently assessed each TSCT scan and determined the type of each CT bronchus sign (A, B, or C). When the two bronchoscopists recommended different types, the final type was determined by discussion.

### Bronchoscopic procedures

After administration of local pharyngeal anesthesia, all patients were lightly sedated with individually calculated dose of intravenous midazolam as reported elsewhere [11]. Transbronchial biopsy (TBB) using EBUS with a guide sheath (EBUS-GS) was performed according to the standard Kurimoto method [12]. A 20-MHz radial type ultrasound probe with an external diameter of 1.4 mm (UM-S20-17S; Olympus Medical Systems, Tokyo, Japan) connected to an endoscopic ultrasonography system (EU-M30S; Olympus Medical Systems) was used in all cases. We mainly used a bronchoscope with a 2.0-mm-diameter working channel (BF-P260F; Olympus Medical Systems) for a guide sheath with an external diameter of 1.9 mm (K-201 kit equipped with a biopsy forceps and cytological brush; Olympus Medical Systems). According to the size or location of the lesion, the operator could use a 1T260 bronchoscope with a 2.6-mm-diameter working channel with the guide sheath kit K203 (external diameter of the guide sheath: 2.55 mm) (Olympus Medical Systems). We took 12 biopsy samples when using the K201 kit and nine samples using the K203 kit. Nine bronchoscopists performed the procedures in this study. Five of them had less than 7 years of experience as a bronchoscopist (range: 2–6 years), whereas four had more experience (range: 7–21 years).

### Diagnostic evaluation of TBB results (positive diagnostic criteria by bronchoscopy)

In this study, we evaluated the bronchoscopic diagnostic yield in both malignant and non-malignant diseases (including inflammatory disease). Thus, we determined the evaluation criteria for judgment of successful (diagnostic) bronchoscopy according to previous reports [8, 13]. Lesions with evident malignant findings on histological examination or class IV/V findings on cytological examination were defined as malignant. For benign lesions, when bronchoscopy demonstrated a distinct histologic pattern (such as epithelioid granuloma or intra-alveolar organization) or the presence of bacteria accompanied by reasonable radiologic and clinical findings, we determined that the bronchoscopy was successful. All benign lesions were followed for more than 12 months to confirm that the lesions had remained stable or improved by appropriate therapy.

### Statistical methods

Statistical analyses were carried out using JMP software (ver. 8.0; SAS Institute, Inc., Cary, NC). A binomial proportion confidence interval (CI) was calculated for diagnostic yield. Differences in proportions were evaluated with the *χ*^2^ test. Spearman rank correlation was used to identify the association between two ordinal variables. Multivariate logistic regression analysis was used to identify factors associated with diagnostic yield independent of other variables. We entered all variables that were significantly (*p* < 0.05) associated with diagnostic yield in the univariate analyses into the multivariate analysis. The final model was determined by a backward variable selection method, and odds ratios and the 95 % CIs are presented. A *p*-value of <0.05 was considered to indicate statistical significance. All analyses were two-sided.

## Results

### Patient characteristics and details of lesions

We retrospectively reviewed 149 consecutive patients who underwent TBB with EBUS-GS. Table 1 shows the characteristics of the studied patients and targeted lesions. The median age of the patients was 70 years (range: 32–86 years). There was no sex difference. The size of the lesions ranged from 7.5 to 29.7 mm, and the median size was 19.6 mm. Five patients (3.4 %) had lesions smaller than 10 mm in diameter. Seventy-seven lesions (51.7 %) were located in the upper lobe and 17 (11.3 %) were located in superior segment of the lower lobe. Twenty-one cases (14.1 %) exhibited ground glass opacity (GGO), including 17 cases with pure GGO lesions. The number of bronchial branches that reached the target lesion from the trachea ranged from 3–8 (median: 5). Chest X-ray and TSCT were assessed as radiological findings evaluable before the decision regarding whether to perform bronchoscopy. On the initial chest X-rays, the targeted lesions were clearly visible in 77 patients (51.7 %) and vague or invisible in 72 patients (48.3 %). After careful assessment of chest TSCT by two bronchoscopists, we determined the type of CT bronchus sign of each lesion: A (86 lesions, 57.8 %), B (49 lesions, 32.9 %), and C (14 lesions, 9.3 %) (Table 1, Fig. 1). The median bronchoscopic examination time was 55 min (range: 43–71 min). The mean total dose of lidocaine used was 345 mg (range: 240–550 mg). Ten patients (6.7 %) had complications including five with pneumothorax requiring pleural drainage, four with pneumonia and one with a transient delirium. No severe complications occurred during this study. Final diagnosis of targeted lesions are shown in Table 2.

**Table 1:** Characteristics of Patients and Targeted Lesions (*n* = 149)

Variable (patients)	
Age	
yrs. median (range)	70 (32–86)
Gender	
Male, number (%)	86 (57.7 %)
Female, number (%)	63 (42.3 %)
Variable (lesions)	
Size of the lesion (diameter)	
mm, median (range)	19.6 (7.5–29.7)
Location of the lesions	
Upper lobe	77 (51.7 %)
Lingular lobe or middle lobe	19 (12.8 %)
Superior segment of the lower lobe	17 (11.3 %)
Lower lobe (except for superior segment)	36 (24.2 %)
Chest X-ray findings of the lesions	
Clearly visible	77 (51.7 %)
Vague or invisible	72 (48.3 %)
Thin slice CT features	
GGO	21 (14.1 %)
Solid	128 (85.9 %)
Number of the branch reach to the lesion	
mean	5.03
3	12 (7.9 %)
4	32 (21.5 %)
5	55 (36.9 %)
6	35 (23.6 %)
7	12 (8.1 %)
8	3 (2.0 %)
CT bronchus sign ^a^	
A	86 (57.8 %)
B	49 (32.9 %)
C	14 (9.3 %)

*GGO* ground glass opacity, ^a^CT bronchus sign: see Methods

**Table 2:** Final diagnosis of targeted lesions (*n* = 149)

Malignancy (*n* = 110)	
Lung cancer	
Adenocarcinoma	72
Squamous cell carcinoma	14
Large cell carcinoma	2
NSCLC	4
Adenosquamous	1
Small cell carcinoma	4
Metastatic cancer	12
Lymphoma	1
Non-malignancy (*n* = 39)	
NTM	10
Organizing pneumonia	10
Non-specific inflammation	6
Fungal infection	3
Benign tumor	2
Bacterial pneumonia	2
Sarcoidosis	2
Tuberculosis	2
Others	2

*NSCLC* non-small cell lung cancer, not otherwise specified, *NTM* non-tuberculosis mycobacteriosis. Others include pulmonary infarction and asbestos-related fibrosis

### Factors associated with bronchoscopic diagnosis

**Fig. 2:**
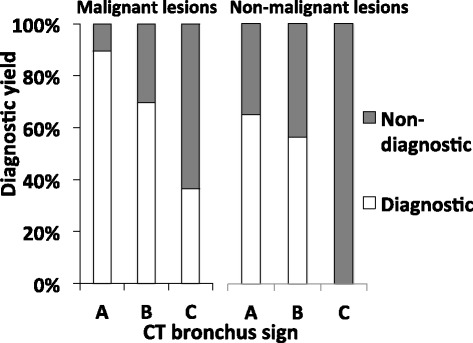
CT bronchus sign and bronchoscopic diagnostic yield. The CT bronchus sign was a significant factor associated with a positive bronchoscopic diagnosis. In particular more than 90 % of malignant lesions with a CT bronchus sign type *A* could be diagnosed successfully. Conversely, in benign lesions, the bronchoscopic diagnostic yield was 0 % in lesions with a CT bronchus sign type *C*

In total, 72.5 % (95 % CI: 64.8–79.0) cases could be diagnosed with TBB using EBUS-GS. The diagnostic yield of malignant diseases and non-malignant diseases was 78.2 % and 56.4 %, respectively (Table 3). The diagnostic yield was significantly higher in malignant than in nonmalignant diseases (*p* = 0.0089). We examined clinical factors that can be obtained at the outpatient clinic for their potential ability to predict the bronchoscopic diagnostic yield. Lesion diameter, lesion visibility on chest X-ray, and the CT bronchus sign were significantly associated with the diagnostic yield in the univariate analyses (Table 4). In the logistic regression analysis, the CT bronchus sign was the only significant factor associated with successful achievement of a bronchoscopic diagnosis. The diagnostic success rate for lesions with a CT bronchus sign type A was 11.1 times higher than that for lesions with a CT bronchus sign type C (Table 5). More than 90 % of malignant lesions with a CT bronchus sign type A could be diagnosed successfully. Conversely, the bronchoscopic diagnostic yield was extremely low in lesion with a CT bronchus sign type C (36.4 % and 0.0 % in malignant and non-malignant lesions, respectively) (Fig. 2).

**Table 3:** Diagnostic yield (all cases)

Diagnostic yield		
All cases	72.5 % (108/149)	
Malignancy	78.2 % (86/110)	
Non-malignancy	56.4 % (22/39)	*p* = 0.0089*

*Significant difference between malignancy and benign lesions

**Table 4:** Contribution of clinical factors available before bronchoscopy to diagnostic yield (all cases)

Variables		Diagnostic yield	*p* value
Lesion diameter	<20 mm	51/80 (63.8 %)	0.01*
	≥20 mm	57/69 (82.6 %)	
Lesion location	Upper lobe or sup segment of lower lobe	40/56 (71.4 %)	0.82
	Middle or lower lobe ^a^	68/93 (73.1 %)	
Feature of the lesion	GGO	12/18 (66.7 %)	0.24
	Solid	96/131 (73.2 %)	
Visibility of the lesion on Chest X-ray	Clearly visible	59/72 (81.9 %)	0.01*
	Vague or invisible	49/77 (63.6 %)	
Number of bronchial branch to reach the lesion	≥5	35/50 (70.0 %)	0.63
	≤4	73/99 (73.7 %)	
Operator’s experience (years)	≥7	34/52 (65.4 %)	0.16
	≤6	74/97 (76.3 %)	
CT bronchus sign^b^	A	72/86 (83.7 %)	0.001*
	B	32/49 (65.3 %)	
	C	4/14 (28.6 %)	

* significant difference, ^a^ except for superior segment of the lower lobe, ^b^ See Methods

**Table 5:** Logistic regression analysis of factors on diagnostic yield of TBB using EBUS-GS

Variables		HR	(95%CI)	*p* value
Visibility on Chest X-ray	Clearly visible	2.03	0.90–4.59	*P* = 0.087
	Vague or invisible	Ref.		
CT bronchus sign^a^	A	11.1	2.99–41.2	*P* < 0.001*
	B	4.62	1.24–17.2	*P* = 0.023*
	C	Ref.		

* significant difference, ^a^ See Methods. *Ref*. reference

### Diagnostic yield according to the combination of CT bronchus sign with chest X-ray findings and lesion size

In the daily clinical setting, we must decide whether a patient should undergo bronchoscopy at the initial evaluation in the outpatient clinic. Informed consent for bronchoscopy should include the probability of establishing a pathological diagnosis. We evaluated the diagnostic yield of bronchoscopy according to the combination of a CT bronchus sign, lesion visibility on the initial chest X-ray, and lesion size (Table 6). When target lesions with a CT bronchus sign type A were clearly visible on chest X-ray, the diagnostic yield for the lesions was high (88.0 % for all lesions and 98.6 % for malignant lesions). The diagnostic yield for malignant lesions with both a CT bronchus sign type A and clear visibility on chest X ray was extremely high (100.0 % for lesions 20–30 mm in diameter and 94.6 % for those <20 mm diameter). When the target lesions showed a CT bronchus sign type B accompanied by vague or invisible chest X-ray findings, the diagnostic yield was around 60 %. In five patients with lesions of <10 mm in diameter (range: 7.5–9.9 mm) (type A in two patients and B in three patients), a bronchoscopic diagnosis could be obtained in both patients with the type A sign. In 17 patients with pure GGO lesions, the CT bronchus signs were A in 10 lesions, B in 5 lesions and C in two lesions. The diagnostic yield was 80 % in type A, 60 % in B, and 0 % in C.

**Table 6:** Diagnostic yield according to the CT bronchus sign, Chest X-ray findings and lesion size

CT bronchus sign	Visibility on Chest X-ray	Lesion size	Diagnostic yield % (total/malignancy)
A	Clearly visible	All size	88.0/98.6
		≥20 mm	91.9/100
		<20 mm	76.9/94.6
	Vague or invisible	All size	77.8/82.8
		≥20 mm	83.3/83.3
		<20 mm	75.0/82.4
B	Clearly visible	All size	68.8/90.9
		≥20 mm	87.5/83.3
		<20 mm	60.0/100
	Vague or invisible	All size	63.6/59.1
		≥20 mm	50.0/50.0
		<20 mm	65.2/64.3

### Relationship between CT bronchus sign and EBUS findings

**Fig. 3:**
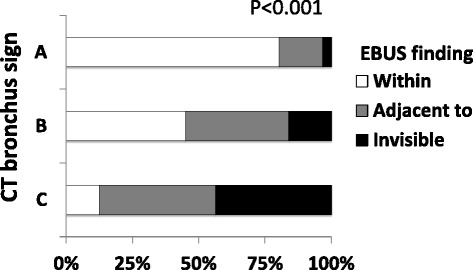
Relationship between CT bronchus sign and EBUS findings. The CT bronchus sign was highly correlated with the EBUS findings Spearman rank correlation coefficient *r* = 0.556, *p* < 0.001. Nearly 70 % of lesions with a CT bronchus sign type *A* showed a “within” finding on EBUS

Previous reports have emphasized the role of the EBUS findings of the target lesion for successful bronchoscopic diagnosis [12, 14]. When an EBUS probe is located in the center of (“within”) the target lesion, the probability of successful diagnosis would be higher than when the probe is located next to (“adjacent to”) or outside (“invisible”) the target lesion. Thus, we evaluated the relationship between the CT bronchus sign and EBUS findings in each case. As shown in Fig. 3, the CT bronchus sign and EBUS findings were well correlated with each other (Spearman rank correlation coefficient *r* = 0.556, *p* < 0.001). Nearly 70 % of lesions with a CT bronchus sign type A showed a “within” finding on EBUS.

## Discussion

It is clinically important to determine which diagnostic modality would be the most reliable and safest for patients with unknown peripheral lesions in the outpatient clinic. Although recent advances in bronchoscopic technologies, including EBUS-GS, have improved the diagnostic yield of bronchoscopy, other modalities such as CTNAB or surgical biopsy might be preferable for some patients. However, these procedures may also be associated with specific complications. Notably, CTNAB is often complicated by pneumothorax and occasionally more serious complications such as air embolism or tumor dissemination along the biopsy route [15]. Thus, it is sometimes difficult to recommend the most suitable diagnostic method to a patient at the initial evaluation. Our study shows that the CT bronchus sign as evaluated by TSCT is the most powerful predictive factor for successful bronchoscopic diagnosis of small peripheral lesions. The physician should encourage the patient to undergo bronchoscopy if the target lesion exhibits a CT bronchus sign type A. On the contrary, if the patient’s lung lesion shows a CT bronchus sign type C, the patient should be offered an alternative diagnostic procedure, such as CTNAB or surgical biopsy.

In this study, we assessed all patients’ TSCT images as their radiological findings at their initial evaluation. However, because it may be difficult to perform TSCT for every patient at some institution, our results might only be valid for bronchoscopy performed at medical centers or educational hospitals in which TSCT is readily available. Future studies are warranted to assess whether conventional CT findings can also be used to predict the results of bronchoscopic biopsy. Our retrospective analysis may also indicate that the differential bronchoscopic diagnostic yield can be determined according to the combination of the CT bronchus sign and chest X-ray findings. This would be useful information that could be offered to patients as a standard method by which to estimate the diagnostic yield of TBB with EBUS-GS. However, these issues must be confirmed in a future prospective study.

Other reports have emphasized that EBUS findings are the most important factor for obtaining pathological results in bronchoscopy with EGUS-GS [12, 14]. These reports have shown that an EBUS finding of “within,” which means that an EBUS probe can be introduced inside the lesion, is strongly related to a high diagnostic yield. In the present study, we demonstrated that a CT bronchus sign type A was significantly related to a “within” EBUS finding. These results might indicate that we can predict EBUS findings from the CT bronchus sign on TSCT at the initial evaluation. Most recently, Evison et al. [16] reported that the presence of a bronchus sign on CT was the only independent predictor of all predefined outcomes, including lesion identification with radial EBUS, positioning of the probe within the center of the lesion, and accurate pathological diagnosis. However, they evaluated larger lesions (nearly 70 % of lesions were >30 mm in diameter) than those in the present study (all lesions were <30 mm in diameter). Our study cohort included five patients with lesions of <10 mm in diameter and 17 with pure GGO lesions. Although the diagnostic yields in these cases were lower than in other cases, a CT bronchus sign type A was still well correlated with a higher diagnostic yield. Taken together, our results clearly indicate that a CT bronchus sign is a powerful predictor of successful bronchoscopy, even in smaller lung lesions.

Our study has some limitations. First, this was a retrospective analysis in a single institution. Because the diagnostic yield may vary among institutions, the usefulness of the CT bronchus sign should be confirmed in a multicenter prospective study. Notably, in the present study, young bronchoscopists with less than 7 years of experience performed about two-thirds of all procedures, and the experience level of the examiner was not related to the diagnostic yield. We speculated that the usefulness of the CT bronchus sign may be universal and independent of differences among institutions or bronchoscopists. Second, it has been reported that the ability to obtain a fluoroscopic view of the lesion significantly influences the success of a bronchoscopic diagnosis. However, a fluoroscopic view of the target lesion is usually difficult to obtain at the patient’s first visit to an outpatient clinic. Therefore, we assessed the chest X-ray findings of the target lesions at the initial evaluation in this study. Third, we did not use a virtual navigation system or rapid on-site examination of the cytological findings. Both of these methods are well known to improve the bronchoscopic diagnostic yield even when using the EBUS-GS system [17–19]. However, we also showed a higher diagnostic yield than those reported in previous studies that used a virtual navigation system and/or rapid on-site examination. Based on the findings of this study, we can say that the CT bronchus sign is an important and useful predictive marker with or without the use of virtual technology or on-site cytological evaluation.

## Conclusions

In summary, the CT bronchus sign associated with target lesions on TSCT is a useful predictive marker for successful bronchoscopic diagnosis of small peripheral lung lesions. Physicians should perform TSCT and evaluate the CT bronchus sign associated with the target lesion before deciding whether bronchoscopy is suitable for the patient.

## Abbreviations

CT: Computed tomography
EBUS-GS: Endobronchial ultrasonography with a guide sheath
TBB: Transbronchial biopsy
CTNAB: Computed tomography guided needle aspiration biopsy
TSCT: Thin-section computed tomography
GGO: Ground glass opacity
CI: Confidence interval
